# European reference network for rare vascular diseases (VASCERN) consensus statement for the screening and management of patients with pathogenic *ACTA2* variants

**DOI:** 10.1186/s13023-019-1186-2

**Published:** 2019-11-21

**Authors:** Ingrid M. B. H. van de Laar, Eloisa Arbustini, Bart Loeys, Erik Björck, Lise Murphy, Maarten Groenink, Marlies Kempers, Janneke Timmermans, Jolien Roos-Hesselink, Kalman Benke, Guglielmina Pepe, Barbara Mulder, Zoltan Szabolcs, Gisela Teixidó-Turà, Leema Robert, Yaso Emmanuel, Arturo Evangelista, Alessandro Pini, Yskert von Kodolitsch, Guillaume Jondeau, Julie De Backer

**Affiliations:** 1 Department of Clinical Genetics and Cardiology and VASCERN HTAD European Reference Centre, Erasmus MC, University Medical Center Rotterdam, Wytemaweg 80, P.O. Box 2040, 3000 CA Rotterdam, The Netherlands; 2 VASCERN HTAD European Reference Centre, Ghent, Belgium; 3 Center for Inherited Cardiovascular Diseases and VASCERN HTAD European Reference Centre, IRCCS Foundation Policlinico San Matteo, Pavia, Italy; 4 Center of Medical Genetics and VASCERN HTAD European Reference Centre, University Hospital of Antwerp University of Antwerp, Antwerp, Belgium; 5 Department of Clinical Genetics and Cardiology and VASCERN HTAD European Reference Centre, Radboud university medical center, Nijmegen, Netherlands; 6 Department of Clinical Genetics and Department of Molecular medicine and Surgery and VASCERN HTAD European Reference Centre, Karolinska University Hospital, Karolinska Institute, Stockholm, Sweden; 7 VASCERN Patient Group (ePAG) and Swedish Marfan organization and VASCERN HTAD European Reference Centre, Färjestaden, Sweden; 8 Department of Cardiology, and VASCERN HTAD European Reference Centre, Academic Medical Center, Amsterdam, Netherlands; 9 Heart and Vascular Center and VASCERN HTAD European Reference Centre, Semmelweis University, Budapest, Hungary; 10 Regional Tuscany Reference Center for Marfan Syndrome and related disorders and VASCERN HTAD European Reference Centre, Careggi Hospital, University of Florence, Florence, Italy; 11 Servei de Cardiologia and VASCERN HTAD European Reference Centre, Hospital Universitari Vall d’Hebron, CIBER-CV, Barcelona, Spain; 12 South East Thames Regional Genetics Service and VASCERN HTAD European Reference Centre, Guy’s Hospital, London, UK; 13 Centro Malattie Rare Cardilogiche – Marfan Clinic and VASCERN HTAD European Reference Centre, Azienda Socio Sanitaria Territoriale Fatebenefratelli – Sacco Milan, Milan, Italy; 14 Department of Vascular Medicine, Department of General and Interventional Cardiology and VASCERN HTAD European Reference Centre, University Heart Center Hamburg, University Medical Center Hamburg-Eppendorf, Hamburg, Germany; 15 CRMR Marfan Syndrome and related disorders, and VASCERN HTAD European Reference Centre Service de cardiologie, AP-HP, Hôpital Bichat-Claude Bernard, Paris, France; 16 INSERM U1148 LVTS and VASCERN HTAD European Reference Centre, Université Paris, Paris, France; 17 Department of Cardiology and Center for Medical Genetics Ghent and VASCERN HTAD European Reference Centre, Ghent University Hospital, Ghent, Belgium

**Keywords:** Thoracic aortic aneurysm, Aortic disease, Dissection, Genetics, Expert testimony

## Abstract

The *ACTA2* gene encodes for smooth muscle specific α-actin, a critical component of the contractile apparatus of the vascular smooth muscle cell. Pathogenic variants in the *ACTA2* gene are the most frequently encountered genetic cause of non-syndromic hereditary thoracic aortic disease (HTAD). Although thoracic aortic aneurysm and/or dissection is the main clinical manifestation, a variety of occlusive vascular disease and extravascular manifestations occur in *ACTA2*-related vasculopathy. Current data suggest possible mutation-specific manifestations of vascular and extra-aortic traits.

Despite its relatively high prevalence, comprehensive recommendations on the care of patients and families with pathogenic variants in *ACTA2* have not yet been established. We aimed to develop a consensus document to provide medical guidance for health care professionals involved in the diagnosis and treatment of patients and relatives with pathogenic variants in *ACTA2*.

The HTAD Working Group of the European Reference Network for Rare Vascular Diseases (VASCERN) convened to review current literature and discuss expert opinions on clinical management of *ACTA2* related vasculopathy. This consensus statement summarizes our recommendations on diagnosis, monitoring, treatment, pregnancy, genetic counselling and testing in patients with *ACTA2-*related vasculopathy. However, there is a clear need for additional prospective multicenter studies to further define proper guidelines.

**Electronic supplementary material:**

The online version of this article (10.1186/s13023-019-1186-2) contains supplementary material, which is available to authorized users.

## Background

Thoracic aortic aneurysm/dissection (TAAD) represents a vascular condition with life-threatening complications, including aortic dissection or rupture. Depending on the presence or absence of manifestations in other organ systems, TAAD can be subdivided into syndromic and non-syndromic. Heritable thoracic aortic disease (HTAD) refers to thoracic aortic disease caused by a pathogenic variant in a gene that confers a high risk for TAAD.

The *ACTA2* gene (OMIM #102620, ORPHA 91387, 2573, 404,463) encodes for smooth muscle cell specific isoform of alpha-actin. Pathogenic variants in the gene are the most frequently encountered cause of non-syndromic HTAD with detection rates varying between 1,5–21%, probably depending on the inclusion criteria of the screened population [1–11].

*ACTA2-*related vasculopathy is an autosomal dominant condition with aortic aneurysm and risk of dissection as main vascular phenotype, and occasional extravascular traits such as skin (i.e. livedo reticularis) and ocular abnormalities (i.e. iris flocculi). The penetrance of the vascular phenotype is incomplete and age-related: according to the limited data available thus far, children usually do not manifest aortic dilatation/aneurysm. Thus far, some case reports and one comprehensive series comprising 277 *ACTA2* mutation carriers have been reported; the main findings are listed in Table 1 [1, 3, 6, 12–15].

**Table 1:** *ACTA2* clinical features

Main clinical manifestation based on refs [1, 3, 6, 12–15]	
– most frequent presenting feature: acute aortic dissection	
– Stanford type A dissection more common (54% @ median age 36 yrs) than type B (21% @ median age 27 yrs)	
– 15–57% @ aortic diameter < 5 cm (often in peri-partum period)	
– Age dependent occurrence of aortic dissection ○ Rare/absent in children ○ Cumulative risk for aortic event (aortic dissection or surgical aneurysm repair) @85y: 76%	
– aortic events more prevalent in males (62%) than in females (38%); *p* = 0.003	
– Risk aortic event higher with pathogenic variants affecting the Arg179 and Arg258 residue, lower with p.(Arg185Gln) and p.(Arg118Gln) pathogenic variants	
– 6% of pregnancies complicated by aortic dissection (third trimester of shortly after delivery)	
Other clinical features	
– Occlusive vascular disease	
○ Premature coronary artery disease (youngest @28 yrs) ○ Moya-Moya like cerebrovascular disease – more common with pathogenic variant affecting the Arg258 residue	
– Congenital Heart Disease	
○ Persistent ductus arteriosus (10%) ○ Bicuspid aortic valve (4%) ○ Atrial septal defect	
– Iris Flocculi (iris cysts at the pupillary border) – more common with p.(Arg149Cys) variant	
– Livedo reticularis	

Multisystem smooth muscle dysfunction syndrome (MSMD syndrome, OMIM #613834, ORPHA 404463) is a severe phenotype caused by de novo pathogenic variants affecting the Arg179 residue of the *ACTA2* gene and is characterized by aortic and cerebrovascular disease, fixed mydriatic pupils, hypotonic bladder, intestinal hypoperistalsis, pulmonary hypertension and white matter brain anomalies [16–23]. Specific management recommendations have been suggested for MSMD syndrome patients and therefore MSMD is not included in these recommendations [24].

This consensus statement focuses on the diagnosis, monitoring and treatment of patient with *ACTA2*-related vasculopathy including genetic counselling. This document represents a disease-specific, preliminary recommendation that certainly will require adjustment and expansion of contents as scientific and clinical insight evolves.

## Methods

The international *ACTA2* consensus group comprised 21 participants from 15 institutions in 9 countries with a shared interest in improving the care for patients with *ACTA2* mutations. All participants (apart from two cooperating guests) are members of the Heritable Thoracic Aortic Disease (HTAD) working group of the European Reference Network on rare vascular diseases (VASCERN), and includes cardiologists, cardiac surgeons and clinical geneticists and all are experts of the disease for having diagnosed, monitored and treated more than 65 families together. The aim of the working group was to develop consensus recommendations in order to standardize and optimize the care for patients and families with pathogenic variants in the *ACTA2* gene.

Our group evaluated the relevant literature to formulate recommendations. An inventory of nine questions on the most debatable *ACTA2*-related topics was sent to all members of the working group. (Additional file 1: Table S1) The inventory and its recommendations were then discussed at two plenary sessions and one video conference call held between September 2017 and May 2018 and were used to formulate the following European recommendations on *ACTA2*-related vasculopathy. The consensus document was approved by all members of the working group.

## Results

Existing international literature on patients with a pathogenic variant in the *ACTA2* gene are often too general with regard to screening recommendations. The results are constituted of recommendations with regard to screening, monitoring, treatment, life-style advices, preconception and pregnancy management for patients with a pathogenic variant in *ACTA2* (excluding MSDM patients). In addition, recommendations with regard to genetic counselling and testing of index patients and their families are composed.

### Diagnosis and monitoring

At initial diagnosis, we recommend 2D-transthoracic echocardiography (2D-TTE) and complete vascular imaging of thorax and abdomen (neck to pelvis) from the age of 18 years. Imaging has to be performed according to established guidelines for each corresponding technique and obtained values have to be corrected for age, BSA and gender. 2D-TTE is the method of choice in the assessment and follow-up of the aortic root and proximal ascending aorta diameters [25, 26]. Measurements are obtained from the parasternal long axis view at end-diastole according to the leading-edge to leading-edge convention. CT and MRI provide information of the entire aorta and peripheral vessels [27]. CT has the best special resolution but low-dose electrocardiographically gated protocols should be used to decrease radiation exposure. MRI has significant advantages in the follow-up of patients with genetic disorders adding functional information to anatomical data without the burden of ionizing radiation. Aortic root measurement is performed by SSFP cine sequence images and the rest of the aorta by 3D-angiogram. Multiplanar reconstruction of the axial source data can create aortic images in a plane perpendicular to the aortic lumen direction (double-oblique or true short-axis images of the aorta) by CT and MRI. There is general consensus that in both imaging techniques the aortic diameter should be measured with the inner-to-inner convention at end-diastole.

**Fig. 1:**
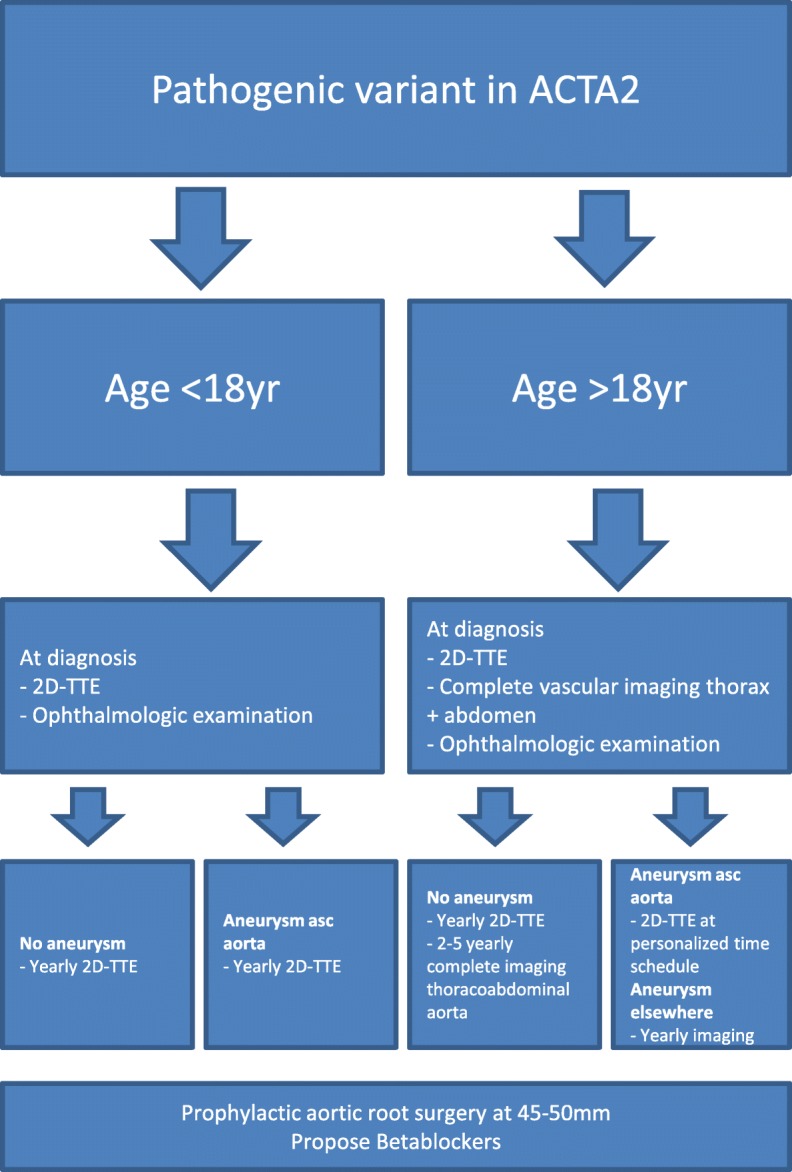
Summary of the screening and management of patients with pathogenic *ACTA2* variants

With an aneurysm of the ascending aorta we recommend 2D-TTE with a time schedule based on the individual characteristics of the patient (age, sex) and the TAA (diameter, growth, shape). With an aneurysm in another artery or in the distal part of the aorta, imaging is recommended yearly, the technique depending on the location of the aneurysm. If no aneurysm is found, enlargement of the ascending aorta is monitored by yearly 2D-TTE of the aortic root and the ascending aorta only. We recommend complete imaging of the vascular tree from neck to pelvis once every two to 5 years (Fig. 1). In children, we recommend yearly 2D-TTE from the age of diagnosis, although intervals can be extended depending on clinical judgement. With normal diameters on echocardiography, computerised tomography angiography (CTA)/magnetic resonance angiography (MRA) imaging of the thoracoabdominal aorta is recommended at the age of 18 years (Fig. 1). The choice for CTA or MRA depends on local expertise and availability, always aiming to keep radiation exposure as low as possible.

No systematic imaging of the cerebral vessels is recommended since there is at present insufficient data to make recommendations for treatment. In patients with symptoms potentially related to cerebral vasculopathy, we recommend imaging according to local guidance and clinical review by Neurology and/or Neurosurgery services. Imaging of the coronary arteries should be limited to patients with symptoms suggesting ischemic heart disease and in patients scheduled for elective aortic surgery.

Ophthalmological examination for detection of iris flocculi is recommended at diagnosis and in patients with symptoms. In young children, the ophthalmological examination can be postponed until they can more easily comply.

### Treatment of aortic aneurysm

Elective aortic root replacement should be considered in asymptomatic patients with a maximal aortic diameter between 45 and 50 mm. The American Heart Association (AHA) recommends a threshold of 45 mm [28] and there is currently no European Society of Cardiology (ESC) recommendation for this topic. Since no additional data have become available, there is no consensus on the threshold. The indications for preventive surgery should consider risk factors such as rate of aortic growth, aortic valve dysfunction, body surface area, family history of aortic dissection, and hypertension. Women planning a pregnancy should be considered for preventive surgery at aortic diameters ≥45 mm (see pregnancy section). For elective intervention, we recommend valve sparing aortic root replacement according to David reimplantation technique of valve sparing root replacement when feasible and in the absence of surgical contraindications such as leaflet fenestrations or relevant aortic valve regurgitation. External aortic support procedures, offered in a limited number of centres, has no established role in this population.

Elective replacement of the descending thoracic aorta should be performed according to the ESC, AHA and European Society for Thoraco Vascular Surgery (ESTVS) guidelines [28–30]. Operative decisions are often difficult and therefore the benefits and risks should be a matter of collective discussion of the patient with the vascular surgeons. There is, at present, no consensus on appropriateness or indications for endovascular procedures in *ACTA2* aortopathy. The general rule is to avoid endoprostheses in patients with genetic aortopathies [29].

### Treatment of aortic dissection

Type A aortic dissection always requires immediate surgical intervention whether or not a pathogenic *ACTA2* variant is present. Valve sparing aortic root replacement should also be considered in this and the risk of coronary artery dissection should be taken into account.

Type B aortic dissections require initial medical management and surgery in case of complications as in patients without *ACTA2* mutations [12]. Endovascular therapy should be avoided in the presence of genetic aortopathies [29].

### Medical treatment

Scientific proof for medical benefit in patients treated with β-blockers is not available. Medications that reduce hemodynamic stress, such as β-blockers, should be considered in *ACTA2* patients, with aortic dilatation (aortic diameter at z-score ≥ 2) or hypertension or progression of aortic dilatation (> 0,3 cm/year) and is reasonable to propose in *ACTA2* patients without dilatation. The use of other antihypertensive drugs can be discussed with the treating clinician.

Hypertension should be treated according to the ESC hypertension guidelines which state that all hypertensive patients with aortic dilatation, should have their blood pressure controlled ≤130/80 mmHg [31]. Patients with a pathogenic variant in *ACTA2* should be managed according to standard strategies for other cardiovascular risk factors, including dyslipidemia [28, 29].

### Lifestyle

Patients should be advised to avoid smoking and substance abuse as for all patients. Endurance sports can be of great value to control body weight, blood pressure and fitness. In patients with aortic dilatation/aneurysm, we recommend low static, dynamic sports such as swimming, walking, running and cycling, without competitive spirit. The physical activity level, both in children and adults, should be adjusted by the cardiologist based on the evaluation of aortic dimensions and valvular function. However, we recommend the avoidance of sports with abrupt isometric or static manoeuvers or any other activities with significant increase in arterial blood pressure, such as weightlifting, competitive football, basketball, handball, and tennis.

### Preconception

For preconception care we recommend counselling of all carriers by a pregnancy heart team with cardiologist (specialized in preconception care), obstetrician, and clinical geneticist for evaluation of the aortic risk, counselling of the risks for the future mother, father and children, including discussion of invasive prenatal diagnostic testing and pre-implantation genetic diagnostics [32]. Imaging of the entire aorta is recommended prior to pregnancy. Women who have a medical history of aortic dissection should be advised not to become pregnant. Women with aortic root diameters ≥45 mm should be advised to undergo prophylactic aortic root replacement prior to pregnancy. Teratogenic drugs (e.g. angiotensin receptor blockers such as losartan and irbesartan) should be discontinued – β-blockers should be considered throughout pregnancy (preferably metoprolol or labetalol).

### Pregnancy management

#### Foetal surveillance

If the genetic status of the foetus is unknown or the foetus carries an *ACTA2* mutation, standardized cardiac ultrasound of the foetus around a gestational age of 20 weeks should be offered due to the slightly increased risk of congenital heart malformations. Many congenital heart malformations associated with *ACTA2* mutations might however be difficult to detect prenatally on ultrasound (e.g. patent ductus, bicuspid valve) [3, 12]. Regular foetal growth monitoring should also be performed in pregnant women who are taking β-blockers because of the associated small risk of foetal growth retardation.

#### Maternal surveillance

Since little data on pregnancies in *ACTA2* mutation carriers are available, we recommend management in pregnant women as for pregnant women with Marfan syndrome [32].

Monitoring (2D-TTE) of the aortic diameter of the pregnant woman with ascending aortic pathology every 4–12 weeks (depending on the severity of dilatation) throughout the pregnancy and 6 months post-partum is recommended.

#### Delivery

In low risk pregnancies (aortic diameter < 40 mm or Z-score < 2 without growth, no aortic dissections in family) the indications and decision for vaginal delivery in hospital should be discussed with the patient who should be aware that, although rare, the risk of dissection during or after delivery may also occur at an aortic diameter lower or around 40 mm [13, 14].

Moderate and high risk pregnancies should be managed by a cardiac and obstetric team with experience in managing patients with aortopathies in an expert center with the option of acute cardiothoracic surgery support. In patients with moderate risk (ascending aortic diameter 40-45 mm), vaginal delivery should be carefully considered on an individual basis. Vaginal delivery should be assisted with expedited second-stage and regional anaesthesia. In high-risk pregnancies (ascending aortic diameter > 45 mm) caesarean delivery should be considered [32].

### Genetic counselling and genetic testing

Genetic counselling should be systematically offered to all patients carrying (or at risk for carrying) a pathogenic variant in *ACTA2*. The counselling aims to collect information and clinical reports of family members, and to inform the consultants of the possible genetic origin of their disease. The genetic work-up should include review with a clinical geneticist who will assess for traits that can potentially recur in patients with *ACTA2*-related vasculopathy as well as other, previously unreported traits. Clinical annotation is fundamental for expanding and collecting observations that may complete the list of the traits of the disease (either markers or abnormalities deserving care).

Cascade genetic testing should be offered in families with probands harbouring pathogenic variants in *ACTA2.* In the case of variants of unknown significance (VUS), in particular novel missense variants, clinical family screening is recommended. Segregation studies in families are essential especially when functional studies are not available or are not feasible.

Each family member that accepts counselling and testing should give his/her informed consent. The counselling process provides the opportunity for informing and discussing current knowledge and data on potential disease-specific risks, and the benefits of genetic testing in order to facilitate autonomous decision-making. Studies of psychological implication of genetic testing for other cardiovascular diseases have shown that the genetic risk is perceived to be manageable {Oliveri, [33] #47}.

Predictive genetic testing of minors is generally accepted for childhood-onset conditions, if preventative or therapeutic measures are available to reduce morbidity or mortality. Although there is no scientific proof for medical benefit in children treated with β-blockers, we advise to propose treatment from the age of 4 years as stated above. Therefore the inclusion of children in the family genetic testing program appears beneficial. The genetic test to evaluate for the *ACTA2* mutation identified in the proband of the family can be performed in the paediatric age using a saliva sample, to avoid the discomfort of blood sampling. In particular, when genetic testing is performed in children under the age of 16 we strongly recommend proper genetic counselling involving psychosocial support.

## Conclusions

Patients affected by *ACTA2*-related vasculopathy will usually be diagnosed due to an incidental detection of aortic dilatation, or after an acute dissection. Coronary or cerebrovascular disease should be assessed in symptomatic patients. Monitoring programs should be tailored to each single patient. Medications such as β- blockers can be considered with the major aim to reduce haemodynamic stress in the aorta. Patients should be carefully screened and monitored for arterial hypertension and treated in order to fully control blood pressure. Preventive aortic surgery should be considered based on general indications, individual needs and risk factors. Since *ACTA2*-related vasculopathy is a recently described condition, the phenotypic spectrum and precise risk factors are probably not fully understood. It is therefore essential to follow these patients at tertiary reference centres, and subsequently report recurring features of the disease. This would enrich the clinical characterisation and facilitate the suspect, thus addressing patients and families to referral centres.

## Additional file

Additional file 1: *ACTA2* clinical features.

## Data Availability

All data generated or analysed during this study are included in this manuscript.

## Abbreviations

2D-TTE: 2D-transthoracic echocardiography
AHA: American heart association
CTA: Computerised tomography angiography
ESC: European society of cardiology
ESTVS: European society for thoraco vascular surgery
HTAD: Hereditary thoracic aortic disease
MRA: Magnetic resonance angiography
MSMD: Multisystem smooth muscle dysfunction
TAAD: Thoracic aortic aneurysm/dissection
VASCERN: European reference network for rare vascular diseases
VUS: Variants of unknown significance
