# Supplementary material for: European reference network for rare vascular diseases (VASCERN) consensus statement for the screening and management of patients with pathogenic ACTA2 variants
Source: Orphanet J Rare Dis. 2019 Nov 21;14:264. doi: 10.1186/s13023-019-1186-2 (PMC6868850; doi:10.1186/s13023-019-1186-2)
Supplement: Supplementary file 1 — ACTA2 clinical features. [file 13023_2019_1186_MOESM1_ESM.docx]

**Table S1**

| **Recommendation in patients with *ACTA2* mutation** | **Strength of recommendation** |
| --- | --- |
| ***Diagnosis and monitoring*** |  |
| The first CTA/MRA of thorax and abdomen in patients with normal diameters on echocardiogram is recommended at the age of 18 years | **++++** |
| The first CTA/MRA of thorax and abdomen in patients with normal diameters on echocardiogram is recommended at the age of 8 years | **+** |
| If no aortic dilatation/aneurysm is found on the first CTA/MRA of thorax and abdomen, the next one is recommended after 2 years | **+++** |
| If no aortic dilatation/aneurysm is found on the first CTA/MRA of the thorax and abdomen, the next one is recommended after 5 years | **+++** |
| ***Prophylactic aortic root surgery*** |  |
| The threshold for elective aortic root aneurysm repair is 45mm | **++++** |
| The threshold for elective aortic root aneurysm repair is 50mm | **+** |
| ***Medical treatment*** |  |
| The preferred medical treatment is β-blockers in all ACTA2 pathogenic variant carriers | **+++** |
| The preferred medical treatment is β-blockers in ACTA2 pathogenic variant carriers with dilated aorta | **+** |
| The preferred medical treatment is β-blockers and ARB in all ACTA2 pathogenic variant carriers | **+** |

Depending on the proportion of votes received, the strength of the recommendation was recorded as follows

+, 1-25% of the votes

++, 26-49% of the votes

+++, 50-75% of the votes

++++, ≥75% of the votes
